# Resveratrol treatment ameliorates hepatic damage via the TGF-β/SMAD signaling pathway in a phenobarbital/CCl_4_-induced hepatic fibrosis model

**DOI:** 10.22038/IJBMS.2024.75737.16398

**Published:** 2024

**Authors:** Merve Aykaç, Eda Balkan, Semin Gedi̇kli̇, Nurinnisa Öztürk

**Affiliations:** 1 Department of Medical Biology, Faculty of Medicine, Ataturk University, Erzurum, Turkey; 2 Department of Histology and Embrylogy, Faculty of Veterinary, Ataturk University, Erzurum, Turkey; 3 Department of Medical Biochemistry, Faculty of Medicine, Ataturk University, Erzurum,Turkey

**Keywords:** Carbon tetrachloride (CCl4), Hepatic fibrosis, Liver, Resveratrol, Transforming growth factor β/small mother against decapentaplegic (TGF-β/SMAD) signalling, SMAD proteins

## Abstract

**Objective(s):**

Liver fibrosis is a wound healing response characterized by excessive accumulation of extracellular matrix proteins. This study aimed to investigate the effects of resveratrol treatment on the TGF-β/SMAD signaling pathway and related biochemical parameters, apoptosis, and liver regeneration phenobarbital-CCl_4_ induced hepatic fibrosis rat model.

**Materials and Methods:**

This model was created through phenobarbital and CCl_4_ (0.2–0.35 ml/kg). Resveratrol (1 mg/kg/day) was administered to the fibrosis and control groups. Immunohistochemical staining was performed to evaluate αSMA, TGF-β1, and PCNA in liver tissue. The TUNEL method and Masson’s Trichome staining were used to determine apoptosis and collagen accumulation. AST, ALP, ALT, total protein, and total bilirubin levels were measured to determine biochemical status. SMAD2, SMAD3, SMAD4, and SMAD7 expression levels were measured to determine TGF-β1 related hepatic fibrosis.

**Results:**

The SMAD2, SMAD3, and SMAD4 mRNA expression levels were increased and the SMAD7 mRNA expression level was decreased in the fibrosis control group. The SMAD7 mRNA expression level was higher in the phenobarbital-CCl_4_ induced resveratrol treated group. Increased biochemical parameters indicating hepatic damage, increased number of apoptotic cells, and collagen accumulation surrounding the central vein were observed in the fibrosis group compared with the other groups. It was concluded that administration of resveratrol ameliorates the adverse effects of hepatic fibrosis by regulating biochemical parameters, controlling TGF-β1/SMAD signaling, enhancing tissue regeneration, and reducing apoptosis in liver cells.

**Conclusion:**

Resveratrol can be a beneficial option for the prevention of liver damage in a phenobarbital-CCl_4_ induced hepatic fibrosis.

## Introduction

Liver fibrosis is a wound-healing response characterized by an increase in the extracellular matrix (ECM) around the sinusoidal cell layer in the disseminated cavity (1–3). It is a result of the imbalance between ECM synthesis and degradation in the fibrotic matrix (4) and may result in liver cirrhosis and hepatocellular carcinoma. The major causes of liver damage are excessive alcohol use, viral hepatitis B (HBV) and C (HCV) infections, metabolic syndromes caused by diabetes, obesity and insulin resistance, and parasitic infections (5). The process following liver injury includes acute and chronic responses (1). When acute liver damage is not severe, adjacent adult hepatocyte cells may regenerate and become apoptotic and necrotic cells (1). However, if the damage recrudesces, the regenerative process fails and hepatocytes are replaced by extracellular matrix proteins with inflammation (2). During chronic diseases, the ECM component transforms into collagen type I, type III, and fibronectin (6, 7).

CCl_4_ is a hepatotoxin that is frequently used in experimental rodent models of liver fibrosis and cirrhosis. It mimics chronic diseases in humans with toxic damage (2). Acute CCl_4_ administration causes hepatotoxicity due to reactive metabolites produced by cytochrome P-450 enzymes expressed in perivenular hepatocytes. Phenobarbital, a cytochrome P450 inducer, is one of the first therapeutic agents characterized for hepatic drug metabolism (8). CCl_4_-induced rat hepatic fibrosis models with intermittent doses are frequently used because of the histological and hemodynamic similarities to liver diseases in humans (9, 10). Acute damage with repeated administration causes centrilobular necrosis associated with inflammation and hepatic stellate cell activation, increased extracellular matrix, and structural changes (9, 10).

TGF-β superfamily members play a role as multifunctional regulators in many biological processes such as morphogenesis, embryo development, adult stem cell differentiation, immune regulation, wound healing, inflammation, and cancer (11, 12). TGF-β family members (TGF-β1, -2, -3) are induced and activated in fibrotic diseases (13). The release of TGF-β1 by necrotic hepatocytes during liver injury is one of the first signals to activate hepatic stellate cells (HSC). The TGF-β1 signal inhibits HSC apoptosis and stimulates HSCs that overproduce matrix proteins such as fibronectin, and collagen types I, III, and IV (14). TGF-β1 disrupts the production of matrix-degrading proteases and increases the regulation of protease inhibitors such as tissue inhibitors of metalloproteinases (TIMPs) and plasminogen activator inhibitors (15). TGF-β1 stimulates fibrogenesis by inducing matrix production through SMAD3-dependent or non-SMAD-related mechanisms (16, 17). SMAD2 and SMAD3 regulate TGF-β-triggered signal transduction. This activates TGF-β receptor type I kinase, resulting in phosphorylation of TGF-β1, SMAD2, and SMAD3 in the TGF- β/SMAD signaling pathway. Active SMAD2 and SMAD3, which form an oligomeric complex with SMAD4, translocate to the nucleus where target genes including SMAD7 are regulated (18, 19). SMAD2 and SMAD3 are strongly activated in liver fibrosis (20). SMAD3 plays an important role in the signal transduction pathway responsible for fibrosis. Fibrogenic genes (collagens) and markers (αSMA and E-cadherin) are SMAD3 dependent, and SMAD3 binds directly to DNA sequences that regulate these target genes (21, 22). TGF-β induces TIMP-1 by activating SMAD3, thereby inhibiting ECM degradation. Over-expression of SMAD3 inhibits MMP-1 activity in fibroblasts. These results indicate the pathogenic role of SMAD3 in TGF-β-induced fibrosis (23). Resveratrol (3,5,4’ trans trihydroxy stilbene) is a natural phytoalexin found especially in red fruits. In addition to having anti-inflammatory and anti-oxidant properties, it plays an important role in regulating lipid metabolism and preventing cancer (24). It has been reported that resveratrol reduces hepatocyte necrosis and has a protective effect on liver damage by regulating collagen accumulation and lymphocyte infiltration in lipid droplets and interlobular spaces of hepatocytes (25, 26). This study aimed to explain the effect of resveratrol on the TGF-β/SMAD pathway in a phenobarbital-CCl_4_-induced liver fibrosis model in 5-6-week-old male Sprague-Dawley rats.

## Materials and Methods

***Experimental animals***

A total of 32 male Sprague-Dawley rats, 150–200 gr weights and aged 5–6 weeks, were purchased from the Ataturk University Medical Experimental Application and Research Center. The rats were maintained in 50*50*30 cm cages (4 rats per cage) and in a controlled environment with a 12:12 hr light/dark cycle at a temperature of 21 °C±2 °C and relative humidity of 55%±10%. Animal welfare and experimental procedures were performed in accordance with the Guide for the Care and Use of Laboratory Animals and were approved by the Animal Ethics Committee of Ataturk University.

***Chemicals***

Resveratrol was purchased from Glentham Life Sciences, GP2549 (UK), dimethyl sulfoxide (DMSO) from BioFroxx (Germany), phenobarbital from Sigma Aldrich (Germany), and CCl_4_ from J.T. Baker (Fisher Scientific, Schwerte, Germany). Masson’s trichome stain was purchased from Bio Optica (Italy), and the TUNEL kit (ApopTag® Peroxidase In Situ Apoptosis Detection Kit) from (Merck Millipore, Darmstadt, Germany). Formalin, ethyl alcohol, toluene, and all other chemicals for laboratory experimentation were purchased from Merck Millipore (Darmstadt, Germany).

***Experimental design***

The experimental design is outlined in Table 1. The rats were randomly separated into four groups (n=8 per group) as follows: 1) Fibrosis group; CCl_4_ (ml/kg/oral gavage/twice a week, 0.2–0.35 ml/kg), saline (10 IU/day/IP), 2) Fibrosis-resveratrol treated group; CCl4 (ml/kg/oral gavage/twice a week, 0.2-0.35 ml/kg), resveratrol (1 mg/kg/day/ IP), 3) Control group; saline (10 IU/day/ip), and 4) Control-resveratrol treated group; saline (10 IU/day/ IP), resveratrol (1 mg/kg/day/ IP). In the first 2 weeks, phenobarbital (0,5 gr/L) was added to the drinking water of the fibrosis and fibrosis-resveratrol-treated groups. Then, the fibrosis group was treated with CCl_4_ (0.30 ml/kg) through oral gavage twice a week for 10 weeks to induce liver fibrosis. In the fibrosis-resveratrol-treated group, the rats were administered CCl_4_ through oral gavage twice a week for 10 weeks and resveratrol at 1 mg/kg/ip daily. In the control and control-resveratrol treated groups, saline at a volume equal to that of CCl_4_ and resveratrol was given to the rats by gavage and IP injection. At the end of the experiments (at 12 weeks), the animals were sacrificed using ketamine/xylazine anesthesia. Blood samples were withdrawn by cardiac puncture, and serum was prepared and stored at −80 °C until biochemical assay. Half of the liver tissues were stored in 10% neutral formaldehyde for histopathological examination and the other half were stored at −80 °C for real-time PCR analysis. The experimental animals were assigned to groups using the Statistical Package of Social Science (SPSS) program for Windows (Standard version 22). Data were presented as mean ± standard deviation (SD) values. The Tukey test was used in the comparisons between groups. A value of *P*<0.05 was accepted as the level of statistical significance.

***Measurement of biochemical parameters***

Serum was separated from the blood samples. The levels of alanine aminotransferase (ALT), aspartate aminotransferase (AST), alkaline phosphatase (ALP), albumin (ALB), total protein (TP), and total bilirubin (TB) in the serum were detected using an automatic biochemical analyzer (Roche Cobas c702, Roche Diagnostics GmbH, Mannheim, Germany) in Ataturk University Health Research and Application Center, Department of Medical Biochemistry.

***Histological evaluations***

The liver tissue samples were taken under anesthesia and immediately fixed in 10% neutral formalin. After passing through a series of 70–100% ethyl alcohol, the tissues were dehydrated in toluene and embedded in paraffin blocks. Sections 3–5 μm in thickness were cut from the blocks and stained with hematoxylin and eosin (H&E) for tissue morphology and with Masson’s trichome stain for collagen fiber structure, according to the manufacturer’s instructions. All slides were analyzed by two researchers in consensus using a multiheaded light microscope (Nikon Elipse i50). Scoring was applied as described by Kaya-Dagistanli, Tanriverdi, Altinok, Ozyazgan, and Ozturk, (2013) as follows, 0: histologically normal liver tissue, 1: centrilobular necrosis and fatty degeneration, 2: centrilobular and midlobular fatty degeneration, perivenular fibrosis, 3: septal fibrosis, pseudolobule formation, and 4: regenerative nodule formation, cirrhosis. HSCORE analysis was used for histological scoring. The preparations were photographed with a digital microscope camera (Toupcam industrial digital camera ICMOS HD camera-Zhejiang, China).

***Immunohistochemistry assay***

All sections were placed on adhesive (poly-L-Lysin) slides for the immunoperoxidase examination, and a deparaffinized—dehydrated procedure by passing through a xylol and alcohol series was performed. The sections were washed in distilled water for 5 min., then washed with phosphate buffer solution (PBS, pH 7.2) for 5 min, kept in 3% H_2_O_2_ for 10 min, and endogenous peroxidase was inactivated. Then, the tissues were boiled in 1% antigen retrieval (citrate buffer (pH +6.0) ab93678) solution and allowed to cool at room temperature. After washing for 5–10 min in PBS, the sections were incubated for 5 min with a protein block (Lot: PHL659660, Thermo Fisher, USA) compatible with all primary and secondary antibodies to prevent non-specific background staining. After removal of the excess block solution remaining on the tissue sections at the end of the incubation, the primary antibodies (α-SMA antibody, (1:50 dilution, Abbkine), TGF-β1 antibody (1:100 dilution, Abbkine) and PCNA antibody (1:100 dilution, Abbkine)) were dropped onto the sections without washing. In accordance with the primary antibody instructions, after incubation at room temperature for 1 hr or at +4 °C overnight, the sections were washed with PBS again, twice for 5 min, and then incubated with a biotinized secondary antibody (Lot: PHL659660, Thermo Fisher, USA) for 10–30 min at room temperature. The sections were then washed again with PBS and streptavidin-peroxidase for 10–30 min, after which 3-3′ Diaminobenzidine (DAB) chromogen (Lot: HDX47396, Thermo Fisher, USA) was dropped onto the sections and these were washed with distilled water. Between 1–2 min of Mayer’s hematoxylin staining was applied for background staining. The sections were washed with tap water, then passed through graded alcohol and xylol series, covered with a coverslip and examined under a light microscope. In this study, phosphate buffered saline (PBS) was used as a negative control instead of primary antibody, but was not included in the pictures. The intensity of liver immunostaining for α-SMA, TGF-β1 and PCNA antibodies was evaluated semi-quantitatively using the following categories: 0 (no staining), 1+ (weak, but detectable staining), 2+ (moderate or distinct staining), and 3+ (intense staining). For each slide, a histological score (HSCORE) value was calculated as the total percentage of cells stained in each intensity category multiplied by the weighted intensity of the staining, using the formula HSCORE Pi (i + 1), where i represents the intensity score and Pi is the corresponding percentage of cells. Five randomly selected areas on each slide were evaluated under a light microscope (Nikon, Eclipse, i50, Tokyo, Japan), and the percentage of cells at the various intensities within these areas was determined.

***TUNEL assay***

Tissue sections were deparaffinized and rehydrated. Apoptotic hepatocytes were detected in the TUNEL assay using the Apoptosis Detection Kit (ApopTag® Peroxidase In Situ Apoptosis Detection Kit, Merck Millipore, Burlington, MA, USA) according to the manufacturer’s instructions. The percentage of positive-stained cells was calculated and presented as the apoptotic index (apoptotic index =number of positive cells/total number of cells*100%).

***Real-time PCR assay***

The total RNA was extracted from the liver tissue using the NucleoSpin® RNA Kit (Macherey-Nagel 740955) according to the manufacturer’s instructions. The concentration of total RNA was measured with the NanoDrop spectrophotometer (ThermoFisher Scientific, Waltham, MA, USA). cDNA was synthesized using the Script cDNA Synthesis Kit (Jena Bioscience). The housekeeping gene β- actin was used as a reference to calculate fold change in target gene expression. SMAD2, SMAD3, SMAD4, SMAD7 and β-actin primers were measured using quantitative real-time PCR (q-PCR) with the ProbeMaster Kit (Jena Bioscience) following the manufacturer’s instructions. The data were calculated according to the 2^−ΔΔCt ^method.

***Statistical analysis***

All the analyses were performed using IBM SPSS® 22.0. software. One-way ANOVA followed by a Tukey post hoc test was used in multiple comparisons. Conformity of the data to normal distribution was evaluated using the Kolmogorov-Smirnov test. All data were expressed as mean ± standard deviation (SD) values. In all analyses a value of *P*<0.05 was considered statistically significant.

**Table 1:** Experimental design for the phenobarbital-CCl_4_-induced hepatic fibrosis model. Sprague- Dawley rats were separated into 4 groups as control group, control-resveratrol treated group, fibrosis group, fibrosis-resveratrol treated group. The experimental design is explained below

**Experimental groups**	**0.-2. Weeks**	**3.-12. Weeks**
Control Group	Chow diet Tap water	Chow diet tap water Saline 10 IU/day/IP (intraperitoneal)
Control-Resveratrol treated group	Chow diet Tap water	Chow diet tap water Resveratrol 1 mg/kg/day/ IP (intraperitoneal)
Fibrosis group	Chow diet 0.5 gr/L phenobarbital added tap water	Chow diet 0.5 gr/L phenobarbital added tap water CCl4 0.30 ml/kg/oral gavage/twice a week Saline 10 IU/day/IP (intraperitoneal)
Fibrosis-Resveratrol treated group	Chow Diet 0.5 gr/L phenobarbital added tap water	Chow Diet 0.5 gr/L phenobarbital added tap water CCl4 0.30 ml/kg/oral gavage/twice a week Resveratrol 1 mg/kg/day/ IP (intraperitoneal)

**Table 2:** The results of the comparisons of the scores of the hepatic biochemical parameters. *1: Fibrosis resveratrol-treated group vs Fibrosis group

	**Control group** **Mean** **±** **SD**	**Control resveratrol-treated group** **Mean** **±** **SD**	**Fibrosis group** **Mean** **±** **SD**	**Fibrosis resveratrol treated group Mean** **±** **SD**	***P*** ***-*** **value**
ALP (U/L)	323.43±44.25	264.44±47.49	494.88±329.03	240.75±54.36	0,001*^1^
AST (U/L)	163,29±29.92	147±15.17	573±72.27	296.50±198.51	0,001*^1^
ALT (U/L)	58.87±9.86	47.8±9.48	553.25±223.96	193.38±65.64	0,001*^1^
TP (g/Dl)	6.27±0.39	6.5±0.34	6.57±0.22	7.02±0.24	0,001*^1^
ALB (G/Dl)	2.96±0.12	2.87±0.14	2.83±0.16	3±0.36	0,001*^1^
TBIL (mg/dL)	0.12±0.05	0.14±0.03	0.28±0.25	0.5±0.38	0,001*^1^

ALP: Alkaline phosphatase; AST: Aspartate aminotransferase; ALT: Alanine aminotransferase; TP: Total protein; ALB: Albumin; TBIL: Total bilirubin

**Table 3:** The comparisons of the gene expression changes in the TGF- β/SMAD Pathway. a: Control group vs Control resveratrol-treated group, b: Control group vs Fibrosis group, c: Control group vs Fibrosis resveratrol-treated group, d: Control group vs Fibrosis group, e: Control resveratrol- treated group vs Fibrosis resveratrol-treated group, f: Fibrosis group vs Fibrosis resveratrol- treated group

**Genes**	**Control group (n = 8) ** **Mean ± SD**	**Control resveratrol-treated group** **(n = 8) ** **Mean ± SD**	**Fibrosis group** **(n = 8) ** **Mean ± SD**	**Fibrosis resveratrol-treated group** **(n = 8) ** **Mean ± SD**	***P*** **-value**
**SMAD-2**	26.43±0.95	25.66±0.91	33.66±1.14	27.99±0.89	0.402^a^ <0.001 ^b,d,e,f^ 0.022^ c^
**SMAD-3**	24.58±0.77	24.76±0.66	35.29±1.35	27.68±0.78	0.980^a^ <0.001^b,c,d,e,f^
**SMAD-4**	25,15±1,31	25,7±1,07	30,06±0,62	28,04±1,12	0.730^a^ <0.001^b,c,d,e,f^
**SMAD-7**	24,81±1,20	24,65±1,20	30,06±0,85	32,91±0,40	0.983^a^ <0.001^b,c,d,e,f^
**β-Actin**	24,16±1,22	25,33±1,76	24,91±0,94	25,81±1,15	<0.05 ^a,b,c,d,e,f^

TGF-β/SMAD: Transforming growth factor- β/small mother against decapentaplegic

**Figure 1:**
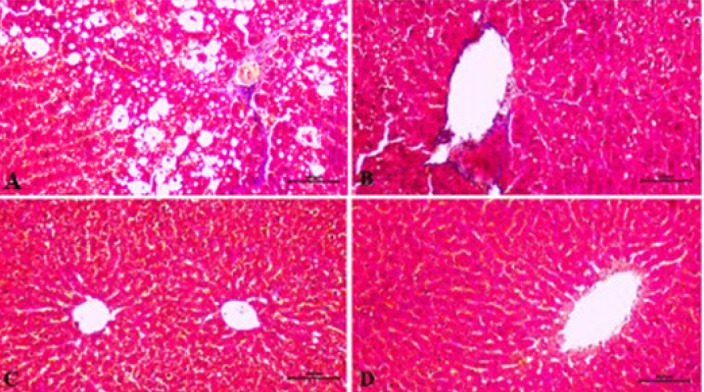
Histopathological examination of liver tissues in various groups of male rats (H&E staining, magnification ×100)

A: Fibrosis group, B: Fibrosis resveratrol-treated group, C: Control group, D: Control resveratrol-treated group

**Figure 2:**
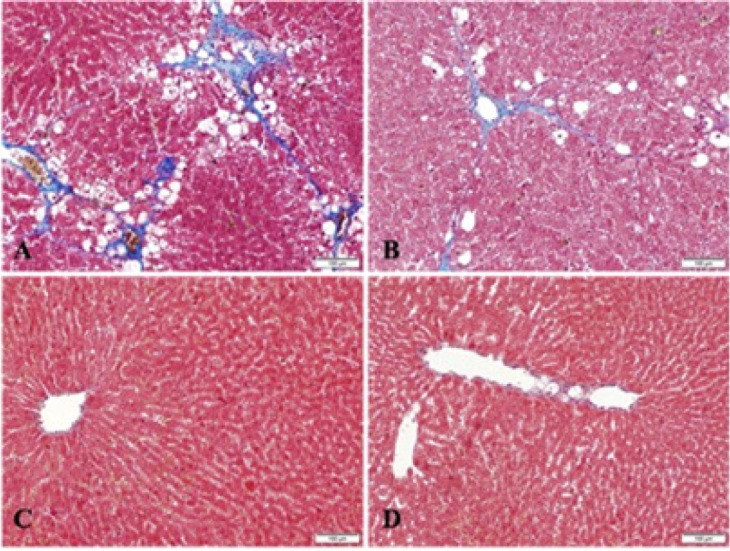
Representative photomicrographs of liver tissues in control and experimental groups of rats (Masson’s trichome stain, x100)

A: Fibrosis group, B: Fibrosis resveratrol-treated group, C: Control group, D: Control resveratrol-treated group

**Figure 3:**
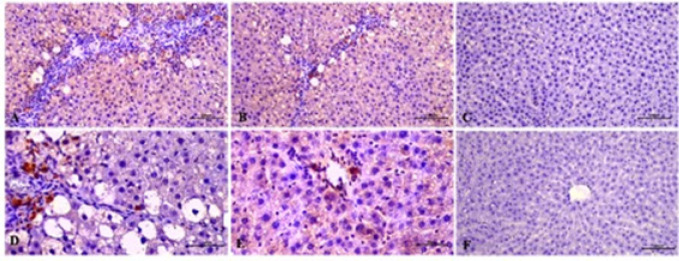
Immunohistochemical staining of αSMA antibody in the control and experimental groups of rats

A: Fibrosis control group (x100), B: Fibrosis resveratrol-treated group (x100), C: Control group (x100), D: Fibrosis group (x200), E: Fibrosis resveratrol-treated group (x200), F: Control resveratrol-treated group (x100)

αSMA: α smooth muscle actin

**Figure 4:**
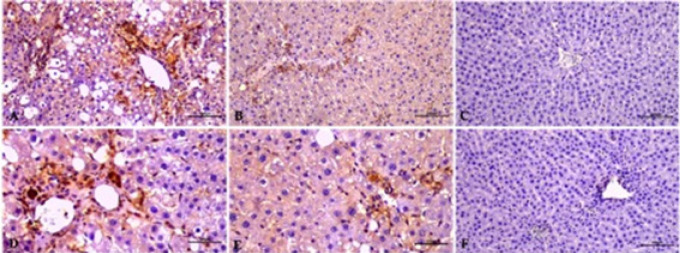
Immunohistochemical staining of TGF-β antibody in the control and experimental groups of rats

A: Fibrosis group (x100), B: Fibrosis resveratrol-treated group (x100), C: Control group (x100), D: Fibrosis group (x50), E: Fibrosis resveratrol-treated group (x50), F: Control resveratrol-treated group (x100)

TGF-β: Transforming growth factor- β

**Figure 5:**
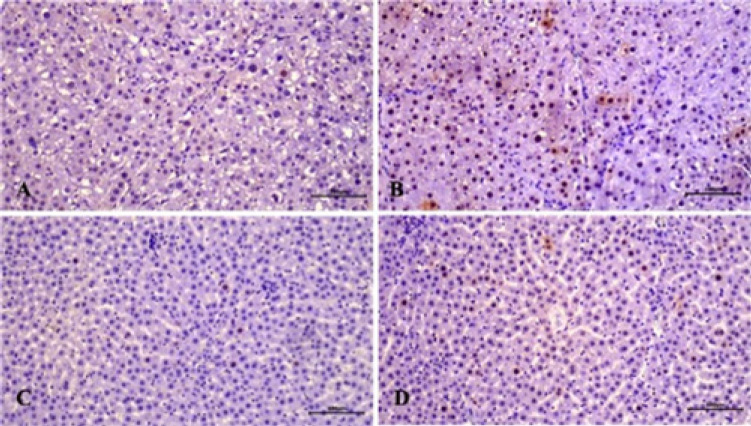
Immunohistochemical staining of PCNA antibody in the control and experimental groups of rats

A: Fibrosis group (x100), B: Fibrosis resveratrol-treated group (x100), C: Control group (x100), D: Control resveratrol-treated group (x100).

PCNA: Proliferating cell nuclear antigen

**Figure 6:**
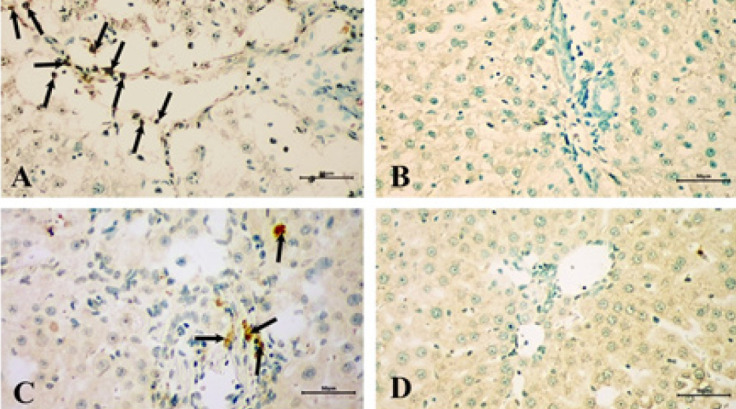
TUNEL staining of liver tissues in the control and experimental groups of rats

A: Fibrosis group (x50), B: Control group (x50), C: Fibrosis resveratrol-treated group (x50), D: Control resveratrol-treated group (x200).

## Results

***Evaluation of liver structure with H*** ***&*** ***E staining***

As a result of the light microscope examinations, it was observed that the histological structure had a normal appearance in the liver sections of the control group (Figure 1C). In the fibrosis group, there was widespread fatty degeneration throughout the entire tissue, fibrosis both around the vena centralis and in the portal area, and severe hydropic degeneration in hepatocytes (Figure 1A). Less vacuolization and degeneration were observed in hepatocytes in the fibrosis resveratrol-treated group compared to the fibrosis group. Fat accumulation around the vena centralis and portal area was also very low (Figure 1B). Morphology similar to that of the control group was observed in the control resveratrol-treated group (Figure 1D).

***Effect of resveratrol on phenobarbital-CCl***_4_ ***induced hepatic collagen changes in liver sections***

Liver tissue sections were stained with Masson’s trichome staining. Collagen distribution was seen to be at a normal level in the control (0.13±0.35) and control resveratrol-treated groups (0.25±0.46) (Figure 2C, D), and intense collagen accumulation was observed in the fibrosis group (2.5±0.53). In addition to the fat accumulation observed within and between the lobules, there was a noticeable increase in the amount of collagen fibers around the vessels and in the portal area (Figure 2A). There was a significant decrease in fat and collagen fiber accumulation in the fibrosis resveratrol-treated group compared to the fibrosis group (1.38±0.52) (Figure 2B).

***Effect of resveratrol on phenobarbital-CCl***_4_ ***induced changes in hepatic biochemical parameters***

The phenobarbital-CCl_4_-induced changes in hepatic biochemical parameters are shown in Table 2. Statistically significant differences between the groups are given in the table. Total protein levels were statistically significantly higher in the fibrosis resveratrol-treated group compared to the other groups (*P*>0.001, Table 2).

***Effect of resveratrol on phenobarbital-CCl***_4_ ***induced gene expression changes in the TGF-β/SMAD pathway***

Phenobarbital-CCl_4_-induced and resveratrol-treated changes in relative SMAD gene expression levels are shown in Table 3 as statistically significant differences between the groups. There was no significant difference between the control and control resveratrol-treated groups in terms of SMAD genes (*P*>0.001, Table 3). There was a significant difference in the fibrosis group compared to the other groups (*P*<0.001, Table 3). The SMAD-2, -3, and -4 gene expression levels were higher in the fibrosis group compared to the other groups. The SMAD-7 expression level was higher in the fibrosis resveratrol-treated group compared to the other groups.

***Protein expression of hepatic α smooth muscle actin (αSMA)***

Cells with stained cytoplasm using αSMA antibody were evaluated as αSMA-immunopositive cells. No αSMA positive cells were seen in the liver sections of the control group (3.00±1.07) and the control resveratrol treated group (4.5±0.93), whereas αSMA immunoreactivity was observed in the control and fibrosis group compared to the other groups (33.38±4.09). Significantly decreased (19.41±1.36) αSMA immunoreactivity was determined in the fibrosis resveratrol treated group compared to the fibrosis group (Figure 3).

***Protein expression of transforming growth factor beta-1 (TGF-β1)***

In the IHC assays, cells with stained cytoplasm using TGF-β1 antibody were assessed as TGF-β1-immunopositive cells. A greater intensity of TGF-1-positive cells was found in the portal area around the central vein in the fibrosis group. Compared to the control and control resveratrol treatment groups, notable TGF-β1-immunreactivity was detected in the fibrosis group (50.2±2.40). No statistically significant difference was found in the comparisons of the control (3.88±0.64) and control resveratrol-treated (4.13±0.83) groups with the fibrosis groups (Figure 4).

***Protein expression of proliferating cell nuclear antigen (PCNA)***

Cells with stained nuclei were evaluated as PCNA-immunopositive cells following IHC staining of the liver tissue sections. Resveratrol administration was seen to stimulate PCNA expression in both the fibrosis and control groups. PCNA-immunopositive cells were scarce in the control (9.85±0.99) and control resveratrol-treated (12.68±1.87) groups. The number of immunopositive cells increased in the fibrosis resveratrol-treated group compared to the fibrosis group (21.83±2.59) (Figure 5).

***Effect of resveratrol on phenobarbital-CCl***_4_ ***-induced hepatic apoptosis***

Apoptotic cells were detected using the TUNEL assay (Figure 6). The TUNEL method shows that labeled nuclei (↑) of apoptotic cells are within the cell cords and the sinusoids in the liver. An increase in the number of TUNEL-positive cells was observed in the fibrosis control group (21.01±2.83) compared to the other groups. A statistically significant reduction of hepatocyte apoptosis was determined in the fibrosis resveratrol-treated group (10.49±1.83) compared to the fibrosis group. The control resveratrol-treated group (2.78± 0.60) was determined to be similar to the control group (2.3±0.44) and the control resveratrol-treated group exhibited no significant alterations in TUNEL positive cells compared to the control group (Figure 6).

## Discussion

Fibrogenesis is a wound healing response to tissue damage. In all acute hepatocellular injuries, fibrogenic pathways are activated. The conversion of normal liver tissue to a fibrotic liver and subsequently cirrhosis is a complex process including various components, primarily hepatic parenchymal and non-parenchymal cells, cytokines, proteases, and protease inhibitors. Several other factors such as age, infection, alcohol intake, and genetic factors have an effect on the rapid progression of fibrosis (27). 

The histological and hemodynamic characteristics seen in a hepatic fibrosis and cirrhosis model developing as a result of repeated doses of CCl_4_ applied to rats are extremely similar to the human fibrosis condition (9, 10). CCl_4_ can be administered orally, subcutaneously, or via inhalation. Hepatotoxicity is caused by reactive metabolites produced by CYP2E1, which is expressed in perivenular hepatocytes as a result of acute CCl_4_ administration. Repeated CCl_4 _administration causes central lobular necrosis associated with an increase in extracellular matrix synthesis as a result of inflammation, hepatic stellate cell activation, and structural changes (9, 10). Moreover, in the long-term administration of CCl_4_, portal hypertension and hepatic decompensation occur. The most commonly used fibrosis model in rats is the phenobarbital-CCl_4_-induced fibrosis model (28). A liver fibrosis model induced by CCl_4_ was developed in male Sprague-Dawley rats aged 5–6 weeks. This model, in which CCl_4_ is applied twice a week, was shown to be more effective than other fibrosis models by Fortea *et al*.(29), who reported that repeated CCl_4_ administration for 12 weeks developed advanced cirrhosis and portal hypertension in rats.

Resveratrol (3,5,4′-trihydroxy-trans-stilbene) is a stilbene group component, found in grape skins and leaves, which is produced by the plant as a response to fungal or bacterial infections, and prevents cellular damage caused by free radicals (24, 30). Resveratrol is known to interact with several specific proteins such as membrane and intracellular receptors, signal molecules, oxidative enzymes, nuclear transcription factors, and DNA repair factors (31). Many *in vivo* and *in vitro *studies have shown the anti-diabetic, anti-oxidant, anti-cancer, and anti-inflammatory effects of resveratrol (24). Proliferating cellular nuclear antigen (PCNA) is a 36 kDa non-histone protein found in the nucleus, which regulates DNA polymerase activity. There is a correlation between PCNA expression and mitotic activity. It has been suggested that resveratrol inhibits Notch-3 and TGF-β proteins by acting as a gamma-secretase inhibitor (DAPT), and inhibits apoptosis in hepatocytes by stimulating hepatocyte proliferation (32, 33). 

In previous studies, resveratrol has been administered long-term (10 weeks) at low doses (0.5–2 mg/kg) or short-term (4 weeks) at high doses (200–400 mg/kg). Ajmo *et al*. reported that high-dose resveratrol treatment had a protective effect against alcoholic fatty liver, while Cho *et al*. stated that low-dose resveratrol was more effective than high-dose (34, 35). In the current study, PCNA expression was observed to be increased in the fibrosis group treated with resveratrol, compared to the fibrosis group. This demonstrated that PCNA could be accepted as a marker of cell proliferation and tissue regeneration and that PCNA expression was increased with the application of resveratrol.

On the liver sections of rats given CCl_4_, liver damage has been determined to be spread over a wide area, and characterized by hepatocellular hydropic degeneration, necrosis around the vena centralis, inflammatory cell infiltration, ballooning degeneration, and expanded sinusoidal spaces (36). In another study, severe fibrosis, apoptosis, and vacuolization were determined in the centrilobular region on liver sections applied with CCl_4_ (37). According to the H&E staining findings in the current study, fibrosis both around the vena centralis and in the portal area, and severe hydropic degeneration in hepatocytes exist. However, the long-term application of low-dose resveratrol was determined to be effective in the healing of the fibrosis and hepatic damage that had occurred. 

Fibrosis is the main characteristic of chronic liver damage. Collagen is the main protein that accumulates in fibrotic tissues (38). A normal liver is formed of an extracellular matrix (ECM) containing glycoproteins such as non-fibrillar type IV collagen, fibronectin, and laminine, and proteoglycans such as heparan sulfate. This matrix, resembling a cage, provides regulation of the molecular signals, which maintain cellular support and cellular differentiation functions (27). Following damage to the liver, there is an increase in the amount of type I and type III collagen fibrils replacing type IV collagen, cellular fibronectin, hyaluronic acid, and other matrix proteoglycans and glycoconjugates in the ECM (27). Chávez *et al*. (38) evaluated hydroxyproline biochemically and collagen fibres with histological staining, and reported that hydroxyproline increased approximately 5-fold with CCl_4_ toxicity, whereas the application of resveratrol reduced collagen accumulation. In another study, the Masson trichrome staining results showed that CCl_4_-induced liver fibrosis in rats caused damage such as deterioration in tissue structure, extension of fibers, formation of large fibrous septa, pseudolobular separation, and collagen accumulation (39). In liver fibrosis models induced with CCl_4_ or dimethylnitrosamine (DMN), resveratrol has been reported to have anti-inflammatory and anti-oxidative effects (38, 40, 41). Resveratrol has been reported to reduce hepatic fibrosis and improve cell vitality with Kupffer cell accumulation in an experimental liver cholestatic injury model (42). In rat livers administered CCl_4_ the number and size of macroscopic nodules were reduced with resveratrol treatment compared to a healthy control group (43). In the current study, the Masson trichrome staining results showed improved tissue morphology with a significant decrease in fat and collagen fiber accumulation in the group administered CCl_4_ and then treated with resveratrol compared to the group to which only CCl_4_ was administered. 

Previous studies have shown that CCl_4_ applied to rats induced liver damage as a result of increasing apoptosis in hepatocytes (44). In the same way, in the rat livers applied with phenobarbital-origin CCl_4_ in the current study, hepatic fibrosis and the number of TUNEL-positive cells were determined to have increased, and resveratrol treatment decreased the number of apoptotic cells. AST, ALT, and ALP are markers of hepatocellular damage. ALT is a cytosolic enzyme of hepatocytes and is an enzyme with increasing activity in plasma membrane permeability associated with cell death (45). Chronic CCl_4_ application increases ALT activity and resveratrol treatment significantly reduces ALT activity (38). Researchers reported that resveratrol significantly reduced AST and ALT levels, the necrosis area, oxidative stress, hepatocyte apoptosis, and the expression of inflammatory markers such as TNF-α and IL-6 (42). In a study, resveratrol was reported to increase the improvement in serum AST, ALT, and ALP levels in a hepatic fibrosis model (46). It has also been reported that repeated CCl_4_ administration significantly increased the serum total bilirubin concentration but normal values were protected with resveratrol treatment (38). Resveratrol has also been shown to have a hepatoprotective effect against acute and chronic liver damage caused by ethanol, methotrexate, and thioacetamidine, as well as CCl_4_ (47, 48). In the current study, the AST, ALT, and ALP values were found to be high in the fibrosis group compared to the fibrosis control group. The T-protein, ALB, and TBIL levels were statistically significantly higher in the fibrosis resveratrol treatment group compared to the other groups, which clearly showed that resveratrol treatment improved phenobarbital CCl_4_-induced liver damage. 

Hepatic stellate cells (HSC) are cells in which vitamin A is stored in the normal liver and contain 40–70% of the retinoid content of the body. HSCs contain type IV collagen in the calm phase but when injury occurs they show phenotypic changes such as decreased retinoid accumulation and cellular proliferation, and increased αSMA (α-actin specific to smooth muscle) and cytokine/chemokine expression in the endoplasmic reticulum. HSC activation plays a central role in hepatic fibrosis (27). Myofibroblasts derived from active HSCs are primarily responsible for collagen and other ECM components produced in the fibrotic liver. There may be an increase in myofibroblasts associated with damage (27). 

A study showed that hepatic damage occurred when the liver was exposed to CCl_4_. A high level of hepatic damage was seen to be associated with high αSMA, which is a marker of HSC activation (49). In the current study, the αSMA immunoreactivity of CCl_4_ toxicity was seen to be increased in the fibrosis group compared with the other groups. The number of αSMA-positive cells was lower in the fibrosis group applied with resveratrol than in the fibrosis group, and this showed that resveratrol could decrease αSMA expression. 

The TGF-β superfamily is formed of structurally and functionally related proteins such as bone morphogenetic proteins (BMP), activin, inhibin, and neurotrophic factors derived from glial (50). These factors participate in many biological processes such as morphogenesis, embryonic development, stem cell differentiation, immune regulation, wound healing, inflammation, and cancer (11, 12).

TGF-β expression occurs in many diseases such as fibrosis and inflammation. It has been reported that multiple tissue lesions developed associated with active TGF- β1 over-expression in the liver of transgenic rats (14, 51). TGF-β1, which has a molecular weight of approximately 25 kDa, is a growth factor with hormone-like activity that can become a dimer form with disulphur bonds (52). TGF-β1 is one of the key mediators in the pathogenesis of hepatic fibrosis (53). The expression of TGF-β1 by necrotic hepatocytes causes HSCs to transform into myofibroblasts. The TGF- β1 signal inhibits HSC apoptosis and induces the synthesis of matrix proteins such as fibronectin and type I, III, and IV collagen (14). TGF-β1 activates SMAD-dependent and independent pathways. While TGF-β has been shown to be associated with the activation of SMAD2 and SMAD3 mediators; it is negatively regulated by the SMAD7 inhibitor (54). When TGF-β is activated, it is bound to the receptor and activates SMADs through phosphorylation. Active SMADs enter the nucleus, are bound to SMAD-binding elements, and regulate the transcription and expression of TGF-β-dependent genes (55). The TGF-β1/SMAD3 signal pathway plays a central role in the pathogenesis of tissue fibrosis (56, 57). SMAD3 is phosphorylated directly by the TGF-β1 receptor serine kinase, and modification after this translation is critical for a profibrotic response-induced TGF-β1/SMAD3 signal (56, 58, 59)

Jang *et al*. showed that resveratrol could inhibit the phosphorylation of SMAD3, which is an important transcription factor for the TGF-β signal, and inhibits fibrogenic gene expression stimulated by TGF-β in an *in vitro *LX-2 cell line (negative human active HSC). It has also been shown that TGF-β causes an increase in Col1A1 level but this effect could be prevented with resveratrol treatment. Resveratrol has been reported to decrease αSMA mRNA and protein levels during HSC activation. It is thought that the anti-fibrogenic effect of resveratrol could be related to SMAD3 inhibition. Although TGF-β increases the phosphorylated SMAD3 level, resveratrol significantly inhibits the SMAD3 phosphorylation stimulated by TGF-β (60). In the current study, the number of immunohistochemical TGF-β1-postive cells was greater in the fibrosis control group than in the other groups. When the TGF-β and SMAD3 mRNA levels were compared, the SMAD3 mRNA levels were determined to be higher in the fibrosis control group. This demonstrated that the administration of resveratrol in addition to repeated phenobarbital CCl_4_ in this rat liver fibrosis model could inhibit SMAD3 mRNA expression, which is a key factor for the TGF-β signal. 

Zhai *et al*. reported that TGF-β1, SMAD2, SMAD3, and SMAD4 mRNA expressions were decreased in the group administered resveratrol compared to the control group, and there was an increase in SMAD7 mRNA expression, which is a SMAD inhibitor (61). In another study of a chronic asthma rat model treated with resveratrol, the phosphorylated SMAD2 and SMAD4 levels were reported to be decreased, but no change was observed in SMAD7 protein expression (62). In the current study, the SMAD2, SMAD3, and SMAD4 mRNA expression levels were determined to be increased, and the SMAD7 mRNA expression level was decreased in the fibrosis control group. A higher SMAD7 mRNA expression level was determined in the group with phenobarbital CCl_4_-induced fibrosis treated with resveratrol. 

## Conclusion

The results of this study demonstrated that the fibrogenic pathways were active in all acute hepatocellular injuries. CCl_4_ is a frequently used hepatotoxin in experimental rodent models of liver fibrosis and cirrhosis. Phenobarbital-origin CCl_4_ triggers hepatic fibrosis in rats. The current study results showed that phenobarbital CCl_4_ changed SMAD expression associated with TGF-β1, hepatic biochemical parameters, and proliferation levels and caused collagen accumulation and apoptosis. The application of resveratrol could be beneficial for the degenerative effects in the liver cells due to CCl_4_. 

## Data Availability

The datasets generated and/or analyzed during the current study are available from the corresponding author upon reasonable request.
